# Combining ability and performance of extra-early maturing yellow maize inbreds in hybrid combinations under drought and rain-fed conditions

**DOI:** 10.1017/S0021859617000636

**Published:** 2017-11-06

**Authors:** I. C. AKAOGU, B. Badu-Apraku, V. O. ADETIMIRIN

**Affiliations:** 1 IITA, 7th floor, Grosvenor House, 125 High Street, Croydon CR0 9XP, UK; 2 Department of Agricultural Biotechnology, National Biotechnology Development Agency, Abuja, Nigeria; 3 Department of Agronomy, University of Ibadan, Ibadan, Nigeria

## Abstract

Maize (Zea mays L.) is a major staple food and cash crop in sub-Saharan Africa (SSA). However, its production and productivity are severely constrained by drought. A total of 120 single-cross hybrids and an open-pollinated control variety were evaluated for 2 years at two locations under managed drought and rain-fed conditions in Nigeria. The objective of the present study was to assess their performance, classify them into distinct heterotic groups and identify promising hybrids for commercialization in the West and Central Africa sub-region. General combining ability and specific combining ability mean squares were highly significant for grain yield and other traits under the research environments. However, there was a preponderance of additive gene action over non-additive. Only six out of 39 inbreds were classified into distinct heterotic groups by the testers. The highest-yielding drought-tolerant hybrid, TZEEI 102 × TZEEI 95, out-yielded the open-pollinated control variety by 43·70%. The average yield reduction under drought was 54·90% of the yield under rain-fed conditions. The hybrids TZEEI 81 × TZEE1 79, TZEEI 100 × TZEEI 63 and TZEEI 64 × TZEEI 79 were the highest-yielding and most stable across environments. These outstanding drought-tolerant hybrids, which are also resistant to *Striga*, have the potential to contribute to food security and increased incomes in SSA and should be tested extensively on-farm and commercialized.

## INTRODUCTION

Maize (*Zea mays* L.) is an important staple food crop in sub-Saharan Africa (SSA) because it is high yielding, easy to cultivate, process and store, and is well-adapted to most agro-ecologies in the region. During the past three decades, the International Institute of Tropical agriculture (IITA) has focused on the development of early (90–95 days to physiological maturity) and extra-early (80–85 days to physiological maturity) maize varieties for production in the savannas of SSA, which are considered the maize belts of the region. The early and extra-early maize cultivars are preferred to traditional cereal crops such as sorghum (*Sorghum bicolor*) and pearl millet (*Pennisetum glaucum*) because they are more responsive to fertilizer application, attain maturity faster and can be harvested much earlier in the season than sorghum and millet crops. Furthermore, the extra-early maize cultivars are used for filling the hunger gap in July in West and Central Africa (WCA) savannas, when all food reserves are depleted after the long dry season and before new crops of the normal growing season are ready for harvest (Badu-Apraku *et al*. 2013*a*). There is also a high demand for early and extra-early maize in the forest agro-ecologies of WCA for peri-urban maize consumers because they allow farmers to market the early crop at a premium price. Furthermore, they are compatible with cassava (*Manihot esculentum*), cowpea (*Vigna unguiculata*) and soybean (*Glycine max*) for intercropping (IITA 1992). Extra-early maize varieties also provide farmers in various agro-ecological zones with the flexibility to plant maize when the rains are delayed as well as when the rainfall distribution is normal (Badu-Apraku *et al*. 2012).

Many improved maize varieties of different maturity have been developed and released by IITA in collaboration with the National Research Systems (NARS) in the WCA sub-region. Despite the availability of improved varieties, the average maize yields in farmers’ fields in WCA is *<*2 t/ha (FAO 2012), largely due to the effects of recurrent drought (Banziger *et al*. 2000). According to Edmeades *et al*. (1995), moisture stress is the major limiting factor to maize production in developing countries and can cause annual yield loss of 24 million tons. The savannas of WCA are severely affected by drought, which can occur at any time during the cropping season. Climate change and its associated effects have also resulted in altered weather patterns, leading to erratic and unreliable amounts and distribution of rainfall, often resulting in drought (Badu-Apraku *et al*. 2013*a*). In maize production, the effect of drought on grain yield is most detrimental at the flowering and grain filling periods (Magorokosho *et al*. 2003). Drought occurrence during the flowering and grain filling periods can cause 22% yield reduction (Hall *et al*. 1981). Up to 90% grain yield reduction has been reported when drought occurs from a few days from before anthesis to the beginning of grain filling (NeSmith & Ritchie 1992).

The focal point of IITA’s Maize Improvement Programme is breeding of cultivars that are *Striga* resistant and tolerant of drought and low nitrogen (N), to increase and stabilize maize production in the WCA sub-region. Two approaches have been adopted for breeding for adaptation to drought. These are the development of extra-early maturing cultivars that complete their life-cycles before severe moisture deficit occurs, i. e. drought escape; and breeding cultivars with better adaptation to drought-prone environments due to the presence of drought-tolerance genes (Badu-Apraku *et al*. 2013*a*, *b*). Drought escape is desirable in cultivars in areas where terminal drought is most prevalent. Adaptation to drought-prone environments, on the other hand, is under genetic control and is an indication of the presence of physiological mechanisms to minimize or withstand the adverse effects of drought when-ever it occurs. Cultivars with enhanced adaptation to drought-prone environments are invaluable where drought occurrence is erratic and with varying intensity (Badu-Apraku *et al*. 2013*a*, *b*).

Grain yield is a complex trait controlled by polygenes, and selection for high grain yield under moisture stress is very difficult due to low heritability of grain yield under stress. As a result, several authors (Banziger *et al*. 2000; Badu-Apraku *et al*. 2012) have proposed the use of secondary traits for selection such as anthesis-silking interval, ears per plant, ear aspect and stay green characteristic, which have high heritability and high correlation with grain yield and are easily measured for selection for high grain yield under moisture stress.

Several *Striga*-resistant and drought-tolerant cultivars with enhanced adaptation to drought-prone environments have been developed by IITA and some of these have been released to farmers after extensive testing in different countries of the sub-region. However, there are only a few commercial extraearly maturing drought and *Striga*-tolerant hybrids in SSA. Information on the combining abilities of inbred lines is important in identifying productive hybrids for commercial hybrid production. Hence, classification of inbred lines that show heterosis under drought is an important step towards addressing the challenges posed by this constraint. This information can be elucidated from studies on heterotic patterns. Separation of maize inbred lines into contrasting heterotic groups is of critical importance in planning crosses and determining the potential of lines for the development of high yielding hybrids. Several studies on heterotic groups under stresses have been carried out for late and intermediate maize (Menkir *et al*. 2003). However, only limited information is available on the heterotic patterns of extra-early inbred lines under drought. Badu-Apraku & Oyekunle (2012) reported significant general combining ability (GCA) and specific combining ability (SCA) mean squares for grain yield and most other traits of seven extraearly inbreds under contrasting environments. The GCA effects were larger than those of SCA in all environments, indicating that additive gene action was more important in the inheritance of the traits. The objective of the present study, therefore, was to determine the yield performance and stability of selected extra-early hybrids with drought-tolerance genes across contrasting research environments for commercialization in WCA and classify the extra-early maize inbred lines into distinct heterotic groups. A companion paper published earlier assessed the genetic diversity of this set of extra-early maturing yellow inbreds and hybrid performance in *Striga*-infested and *Striga*-free environments (Akaogu *et al*. 2012).

## Materials and Methods

A total of 120 extra-early testcross hybrids and an open-pollinated variety, 2008 Syn EE-Y DT STR, used as a control were assessed under (i) managed drought at Ikenne (forest–savanna transitional zone, 6° 87′N,3° 7′E, 60 m a.s.l., 1500 mm annual rainfall) during the dry seasons of 2010 and 2011; (ii) terminal drought at Bagauda (Sudan savanna, 12° 00′N, 8°22′ E, 580 m a.s.l., 800 mm annual rainfall) in 2010; and (iii) rain-fed conditions under which moisture was not a limiting factor at Ikenne during the growing seasons of 2010 and 2011 and Bagauda in 2011. The trials were laid out as an 11 × 11 randomized incomplete block design with two replications. Row and hill spacings were 0·75 and 0·40 m, with two plants/hill, resulting in a population density of 66 666 plants/ha. The managed drought stress at Ikenne was achieved by withdrawing irrigation water 21 days after planting till harvesting, so that the maize plants relied on stored water in the soil for growth and development. Fertilizer was applied to the rain-fed and drought plots at the rate of 60 kg/ha each of N, phosphorus (P) and potassium (K) at planting. An additional 60 kg N was top-dressed at 2 weeks after planting (WAP). The trials were kept weed-free with the application of Atrazine and Gramoxone as pre and post-emergence herbicides at 5 litres/ha each of Primextra and Paraquat, and subsequent hand weeding.

Data collected for each of the experiments were days to silking, days to anthesis (DA), plant height, ear height, husk cover, ear aspect and plant aspect. Anthesis-silking interval and ears per plant were computed as described by Badu-Apraku *et al*. (2011). Leaf senescence data (stay green characteristic) were recorded only for the moisture stressed plots at 65 days after planting on a scale of 1 to 9 where 1 = almost all leaves green and 9 = all leaves dead. Grain yield was adjusted to 15% moisture and computed from the shelled grain weight. In the rain-fed experiments, grain yield was computed based on 80% shelling percentage and adjusted to 15% moisture content.

### Statistical analysis

Analysis of variance (ANOVA) was performed separately for grain yield and other measured traits for the drought stress and rain-fed conditions with PROC GLM in SAS using a RANDOM statement with the TEST option (SAS Institute 2011). Similarly, ANOVA was conducted for all traits across research conditions. In the ANOVA, the location–year combinations, replicates and blocks were considered as random factors, while hybrids (genotypes) were considered as fixed effects and the adjusted means and standard errors were estimated. Means generated from the analysis of variance for each research condition were used for the line × tester analysis as described by Singh & Chaldhary (1985). General combining ability and SCA and their standard errors were computed for grain yield and other traits measured under the two research conditions, using the SAS program.

The hybrid source of variation was partitioned into variability due to lines, testers and line × testers. Estimate of GCA of a tester (male) was obtained based on its performance in *F*_1_ hybrid combinations with all possible lines (females). Similarly, GCA of a line was determined in terms of its performance in *F*_1_ hybrid combinations with all testers. The GCA and SCA effects were determined for traits under each research condition as follows:

$${\text{GCA}}_{j}\;=\;{\bar{y}}_{jk}\;-\;{\bar{y}}_{k},$$

$${\text{GCA}}_{i}\;=\;{\bar{y}}_{jk}\;-\;{\bar{y}}_{k},$$

for line *j* and tester *i*, respectively

$${\mathrm{SCA}}_{ij}=\;{\bar{y}}_{ijk}-\;{\bar{y}}_{ik}-{\bar{y}}_{jk}+\;{\bar{y}}_{k}$$

Where ${\bar{y}}_{ijk},\;{\bar{y}}_{ik},\;{\bar{y}}_{jk},{\bar{\;y}}_{k}$; are the average values for the line by tester, tester, line and research condition, respectively.

The relative significance of GCA and SCA was computed using the method of Baker (1978) as modified by Hung & Holland (2012).

Repeatability of the traits was computed on the hybrid-mean basis (Falconer & Mackay 1996) using the formula:

$$R\;=\;\frac{\sigma_{G}^{2}}{\sigma_{G}^{2}+\left(\sigma_{GE}^{2}/\text{e}\right)+\left(\sigma^{2}/\text{re}\right)}\;$$

Where $\sigma_{G}^{2}$is the genotypic variance, $\sigma_{GE}^{2}$ is the genotypic × environment variance, $\sigma^{2}$ is the error variance, e is the number of environments and r is the number of replications. Variances were estimated using the restricted maximum likelihood (REML) method in SAS MIXED procedure.

The best genotypes under drought were identified based on the index values estimated using the following equation:

I=[(2×Yield)+EPP −ASI - PASP - EASP-SGC]

where EPP is ears per plant, ASI is the anthesis-silking interval, PASP is the plant aspect, EASP is ear aspect and SGC is the stay-green characteristic. Under managed drought, each trait was standardized with a mean of 0 and standard deviation of 1 to minimize the effects of different scales. A positive value therefore indicated the tolerance of the inbred lines to drought, while a negative value indicated susceptibility (Badu-Apraku *et al*. 2011). The best 26 and the worst nine single-cross hybrids under drought and rain-fed conditions were selected using the base index. The yield data of the 35 selected hybrids were subjected to genotype main effect plus genotype × environment interaction (GGE) biplot analysis to decompose the genotype × environment interactions (GEI) under drought stress, rain-fed and across research conditions (Yan *et al*. 2000; Yan 2001). The GGE biplot was used to obtain information on the hybrids that were suitable under drought as well as rain-fed conditions and to investigate the stability of hybrids in the contrasting test environments. The analyses were done using GGE biplot, a windows application that fully automates biplot analysis (Yan 2001). The GGE biplot model equation is as follows:

$$y_{ij}-y_{j}={⋌}_{1}\xi_{\text{i1}}\eta_{j1}+{⋌}_{2}\xi_{j2}\eta_{j2}+\sum_{ij}$$

where *Y_ij_* is the average yield of genotype *Y_i_* in environment *j*, *Y_j_*is the average yield across all genotypes in environment *j*, ʎ_1_ and ʎ_2_ are the singular values for principal component (PC) 1 and PC2, $\xi_{\text{i1}}$ and $\xi_{\text{i2}}$ are the PC1 and PC2 scores for genotype *i*, *η*_*j*1_ and *η*_j*2*_ are the PC1 and PC2 scores for environment *j* and Σ*_ij_* is the residual of the model associated with the genotype *i* in environment *j*. For this, the data were not transformed (‘Transform_0’), not standardized (‘Scale_0’), and were environment-centred (‘Centering_2’).

## RESULTS

Performance of single-cross hybrids under drought and rain-fed conditions

The combined analysis of variance revealed that the genotype (G), environment (E) and their interaction (GEI) mean squares for grain yield and all measured traits were significant (*P* < 0·01) under drought, rainfed and across research conditions except environment mean squares for DA and the anthesis-silking interval and ear height under rain-fed environments (Table 1). The grain yield of the hybrids ranged from 0·46 t/ha for TZEEI 102 × TZEEI 63 to 2·71 t/ha for TZEEI 102 × TZEEI 95 under drought and 1·20 t/ha for TZEEI 102 × TZEEI 63 to 5·38 t/ha for TZEEI 81 × TZEEI 79 under rain-fed conditions (Table 2). The mean grain yield under drought represented 52·3% of the yield in the same environments under rain-fed conditions. Across environments, the average yield reduction under drought was 58·5%. The highest yielding hybrid under drought, TZEI 102 × TZEI 95, and the best hybrid under rain-fed conditions, TZEEI 81 × TZEEI 79, out-yielded the best open-pollinated control variety, 2008 SYN EE Y DT STR, by 43·7 and 30%, respectively.

**Table 1:** Mean squares of grain yield and other agronomic traits of 120 extra-early maize hybrids and an open-pollinated control variety evaluated under drought, rain-fed and across research environments in Nigeria during the 2010 and 2011 growing seasons

Source	DF	Grain yield (kg/ha)	DA	DS	ASI	PLHT	EHT	HC	PASP	EASP	EPP	SGC
Drought Environment (E)	2	342 896 739·2 (*P* < 0·01)	1306 (*P* < 0·01)	86 (*P* < 0·01)	1352 (*P* < 0·01)	74 823 (*P* < 0·01)	3478 (*P* < 0·01)	80 (*P* < 0·01)	19 (*P* < 0·01)	40 (*P* < 0·01)	12·89 (*P* < 0·01)	156·7 (*P* < 0·01)
Genotype (G)	120	839 423·7 (*P* < 0·01)	9 (*P* < 0·01)	26 (*P* < 0·01)	9 (*P* < 0·01)	495 (*P* < 0·01)	284 (*P* < 0·01)	0·3 (*P* < 0·01)	0·5 (*P* < 0·01)	0·6 (*P* < 0·01)	0·04 (*P* < 0·01)	0·7 (*P* < 0·01)
BLK (E × REP)	60	272 585·8 (*P* < 0·01)	5 (*P* < 0·01)	15 (*P* < 0·01)	9 (*P* < 0·01)	673 (*P* < 0·01)	308 (*P* < 0·01)	0·4 (*P* < 0·01)	0·5 (*P* < 0·01)	0·3 (*P* < 0·01)	0·03 (*P* < 0·01)	1·0 (*P* < 0·01)
REP (E)	3	421 600 (*P* < 0·05)	9 (*P* < 0·01)	13 (NS)	4 (NS)	3404 (*P* < 0·01)	2050 (*P* < 0·01)	0·3 (NS)	0·2 (NS)	0·8 (*P* < 0·01)	0·06 (*P* < 0·05)	10·1 (*P* < 0·01)
G × E	238	334 304·5 (*P* < 0·01)	3 (*P* < 0·01)	10 (*P* < 0·01)	7 (*P* < 0·01)	245 (*P* < 0·01)	103 (*P* < 0·01)	0·2 (*P* < 0·01)	0·3 (*P* < 0·01)	0·2 (*P* < 0·01)	0·02 (*P* < 0·05)	0·7 (*P* < 0·01)
Error	298	138 176	11·8	7·4	4·8	170·3	61·6	0·17	0·14	0·17	0·020	0·42
CV		23·50	2·40	4·88	57·48	9·32	12·14	16·96	13·14	13·51	18·52	15·58
Repeatability		0·86	0·74	0·84	0·76	0·82	0·86	0·69	0·83	0·84	0·81	0·72
Optimal Environment (E)	2	326 060 932·50 (*P* < 0·01)	316·3 (*P* < 0·01)	330 (*P* < 0·01)	0·2 (NS)	10 151 (*P* < 0·01)	111 (NS)	21·5 (*P* < 0·01)	8·3 (*P* < 0·01)	5·36 (*P* < 0·01)	0·32 (*P* < 0·01)	
Genotype (G)	120	3 166 774·50 (*P* < 0·01)	6·1 (*P* < 0·01)	10 (*P* < 0·01)	1·5 (*P* < 0·01)	461 (*P* < 0·01)	367 (*P* < 0·01)	0·3 (*P* < 0·01)	0·6 (*P* < 0·01)	0·52 (*P* < 0·01)	0·01 (*P* < 0·01)	
BLK (E × REP)	60	782 208·80 (*P* < 0·01)	2·5 (*P* < 0·01)	4 (*P* < 0·01)	0·7 (*P* < 0·05)	327 (*P* < 0·01)	200 (*P* < 0·01)	0·2 (*P* < 0·05)	0·2 (*P* < 0·05)	0·14 (*P* < 0·01)	0·00 ns	
REP (E)	3	6 193 722·70 (*P* < 0·01)	0·9 (NS)	1 (NS)	1·4 (*P* < 0·05)	1748 (*P* < 0·01)	773 (*P* < 0·01)	0·4 (*P* < 0·01)	0·1 (NS)	1·15 (*P* < 0·01)	0·02 (*P* < 0·05)	
G × E	240	951 188·20 (*P* < 0·01)	1·9 (*P* < 0·01)	3 (*P* < 0·01)	0·8 (*P* < 0·01)	161 (*P* < 0·01)	136 (*P* < 0·01)	0·2 (*P* < 0·01)	0·2 (*P* < 0·01)	0·20 (*P* < 0·01)	0·01 (*P* < 0·01)	
Error	300	435 888·00	0·97	1·4	0·48	78·3	65·0	0·12	0·14	0·080	0·010	
CV		17·32	1·98	2·39	47·68	5·08	10·12	19·48	15·25	9·79	8·04	
Repeatability		0·89	0·88	0·89	0·81	0·87	0·87	0·78	0·85	0·87	0·79	
Across Environment (E)	5	619 872 669 (*P* < 0·01)	1011 (*P* < 0·01)	2220 (*P* < 0·01)	1224 (*P* < 0·01)	118 087 (*P* < 0·01)	17 823 (*P* < 0·01)	73·0 (*P* < 0·01)	25·7 (*P* < 0·01)	19·0 (*P* < 0·01)	9·10 (*P* < 0·01)	
Genotype (G)	120	3 083 694 (*P* < 0·01)	12 (*P* < 0·01)	27 (*P* < 0·01)	7 (*P* < 0·01)	784 (*P* < 0·01)	568 (*P* < 0·01)	0·4 (*P* < 0·01)	0·8 (*P* < 0·01)	0·8 (*P* < 0·01)	0·04 (*P* < 0·01)	
BLK (E × REP)	120	522 705 (*P* < 0·01)	4 (*P* < 0·01)	9 (*P* < 0·01)	5 (*P* < 0·01)	491 (*P* < 0·01)	248 (*P* < 0·01)	0·3 (*P* < 0·01)	0·3 (*P* < 0·01)	0·2 (*P* < 0·01)	0·02 (*P* < 0·01)	
REP (E)	6	3 322 560 (*P* < 0·01)	5 (*P* < 0·01)	8 (NS)	3 (NS)	2573 (*P* < 0·01)	1397 (*P* < 0·01)	0·4 (*P* < 0·05)	0·2 (*P* < 0·01)	0·9 (*P* < 0·01)	0·04 (*P* < 0·01)	
G × E	598	676 934 (*P* < 0·01)	3 (*P* < 0·01)	6 (*P* < 0·01)	4 (*P* < 0·01)	194 (*P* < 0·01)	111 (*P* < 0·01)	0·2 (*P* < 0·01)	0·2 (*P* < 0·01)	0·2 (*P* < 0·01)	0·02 (*P* < 0·01)	
Error	593	289 389	1·3	4·4	2·6	119·4	63·3	0·14	0·14	0·12	0·010	
CV		19·88	2·20	3·94	72·77	6·95	11·02	18·07	14·14	11·83	12·89	
Repeatability		0·96	0·96	0·95	0·89	0·95	0·96	0·88	0·94	0·95	0·91	

DF, degrees of freedom; DA, days to anthesis; DS, days to silking; ASI, anthesis-silking interval; PLHT, plant height; EHT, ear height; HC, husk cover; PASP, plant aspect; EASP, ear aspect; EPP, ears per plant; SGC, stay green characteristic; CV, coefficient of variation; NS, not significant.

**Table 2:** Grain yield and other traits of some hybrids (best 26 and worst 9 based on base index) evaluated under induced drought stress (DT) at Ikenne and terminal drought in Bagauda and under rain-fed conditions (OPT) at Ikenne and Bagauda in 2010 and 2011

Hybrid	Grain yield (kg/ha)		Days to anthesis		Anthesis-silking interval		Plant aspect(scale 1–5)		Ear aspect(scale 1–5)		Ears per plant		SGC	Base Index
	DT	OPT	DT	OPT	DT	OPT	DT	OPT	DT	OPT	DT	OPT		
TZEEI 102 × TZEEI 95	2707	3774	50·8	48·4	2·5	0·3	2·0	2·5	2·5	3·0	0·9	0·97	3·0	15·66
TZEEI 97 × TZEEI 79	2471	4292	49·7	48·4	2·0	1·1	2·4	2·9	2·5	2·7	0·9	0·93	3·5	12·53
TZEEI 71 × TZEEI 79	2253	4241	51·3	49·0	2·5	0·4	2·3	2·3	2·9	2·9	0·8	0·93	3·8	8·26
TZEEI 80 × TZEEI 95	1992	3755	51·3	49·8	2·7	−0·1	2·5	2·7	2·8	3·1	0·9	0·95	3·7	8·17
TZEEI 76 × TZEEI 79	1960	4368	51·2	49·0	2·3	0·4	2·4	1·9	3·0	2·5	0·9	0·96	3·5	8·11
TZEEI 74 × TZEEI 79	2055	3733	49·7	48·5	2·7	0·2	2·5	2·5	2·7	2·9	0·8	0·92	3·5	8·08
TZEEI 61 × TZEEI 95	2434	4150	52·8	48·3	4·5	0·5	2·5	2·9	2·5	2·9	0·8	0·99	4·0	7·75
TZEEI 82 × TZEEI 79	2060	4479	50·5	49·4	2·2	0·3	2·4	2·2	2·6	2·6	0·7	1·00	3·7	7·75
TZEEI 86 × TZEEI 79	2020	4538	51·0	48·8	3·7	0·6	2·6	2·2	2·4	2·5	0·8	0·98	3·7	7·50
TZEEI 100 × TZEEI 63	1913	4937	52·2	50·7	2·3	−0·0	2·3	2·3	2·7	2·5	0·8	0·98	3·8	7·41
TZEEI 67 × TZEEI 79	1906	4441	52·5	50·9	2·2	−0·2	2·4	1·9	2·5	2·4	0·8	0·92	4·2	6·82
TZEEI 98 × TZEEI 63	1982	4888	51·8	48·6	2·7	0·6	2·8	2·1	2·6	2·7	0·9	0·98	3·8	6·60
TZEEI 94 × TZEEI 95	1908	3453	52·3	47·9	2·7	0·4	2·8	2·7	2·5	3·1	0·8	0·98	3·8	6·42
TZEEI 83 × TZEEI 79	1961	4709	50·2	49·3	2·8	0·1	2·4	2·1	2·7	2·6	0·8	1·00	4·0	6·08
TZEEI 73 × TZEEI 79	1878	4491	50·7	49·1	2·8	1·5	2·3	2·1	2·8	2·9	0·9	0·99	4·2	5·95
TZEEI 64 × TZEEI 79	1994	4690	50·7	48·5	2·5	0·2	2·6	2·8	2·8	2·6	0·8	1·01	4·2	5·80
TZEEI 108 × TZEEI 79	1917	4435	51·0	50·3	3·5	1·0	2·4	2·1	2·5	2·8	0·8	1·01	4·3	5·72
TZEEI 71 × TZEEI 95	1981	4388	50·7	48·7	3·0	0·4	2·8	2·2	2·8	2·5	0·8	0·98	3·5	5·64
TZEEI 80 × TZEEI 79	1886	4682	51·7	49·1	3·2	0·6	2·6	2·2	2·8	2·9	0·8	1·04	3·7	5·56
TZEEI 76 × TZEEI 95	1853	3987	51·8	48·3	2·7	0·1	2·8	2·1	2·8	3·0	0·7	0·99	3·3	5·29
TZEEI 59 × TZEEI 79	1922	3513	51·5	49·6	3·5	1·0	2·6	2·3	2·7	2·9	0·8	0·90	4·0	5·26
TZEEI 72 × TZEEI 79	1948	4669	51·2	48·9	3·0	0·2	2·6	2·2	2·8	2·5	0·8	0·99	4·2	5·19
TZEEI 9 × TZEEI 79	1928	3382	49·2	48·9	3·0	0·3	2·5	2·4	2·6	3·1	0·8	0·89	4·3	5·05
TZEEI 98 × TZEEI 95	1665	3626	52·5	50·0	2·3	0·1	2·8	2·5	2·8	2·7	0·8	0·96	3·7	4·79
TZEEI 100 × TZEEI 79	1837	4084	53·0	51·3	2·0	1·3	2·6	2·7	2·9	2·9	0·8	1·03	4·0	4·64
TZEEI 81 × TZEEI 79	2060	5382	52·2	49·8	4·2	0·2	3·0	1·8	2·8	2·3	0·7	0·99	4·3	1·72
CHECK-2008 SYN EE-Y DT STR	1525	3652	51·8	50·7	3·2	0·7	2·6	2·5	3·0	3·1	0·7	0·89	4·2	0·96
TZEEI 61 × TZEEI 63	1111	2551	53·2	52·2	7·0	1·3	3·3	3·0	3·3	3·2	0·7	0·91	5·0	−10·19
TZEEI 58 × TZEEI 63	753	2743	53·2	50·8	6·3	0·7	3·2	2·7	3·7	3·3	0·6	0·95	4·0	−10·38
TZEEI 115 × TZEEI 63	728	2148	53·5	51·2	4·8	1·1	3·5	3·2	3·6	3·5	0·5	0·88	4·5	−11·83
TZEEI 75 × TZEEI 63	901	1834	55·8	50·8	5·5	2·5	3·8	3·2	3·7	3·8	0·5	0·90	4·3	−12·92
TZEEI 76 × TZEEI 63	761	2527	53·2	50·5	7·0	1·5	3·7	3·3	3·7	3·1	0·5	0·95	4·8	−14·99
TZEEI 102 × TZEEI 63	462	1203	55·2	51·7	5·5	1·9	3·8	3·2	3·8	4·1	0·5	0·71	4·3	−15·09
TZEEI 74 × TZEEI 63	728	2178	52·2	50·0	6·3	0·8	3·8	3·0	3·8	3·5	0·6	0·89	5·5	−17·10
TZEEI 62 × TZEEI 63	503	1632	54·3	51·9	8·8	1·9	3·8	2·8	4·1	3·8	0·5	0·75	5·3	−21·25
Means	1581	3812	51·9	49·7	3·8	0·7	2·9	2·5	3·0	8·3	0·7	0·96	4·1	
Standard error (S.E.)	164·1	291·1	0·55	0·43	0·96	0·31	0·17	0·55	0·18	0·65	0·06	0·034	0·28	

SGC, stay green characteristic.

Under drought stress, the repeatability estimates ranged from 0·69 for husk cover to 0·86 for grain yield and ear height, while under rain-fed conditions it ranged from 0·78 for husk cover to 0·89 for grain yield and days to silking (Table 1).

Under drought stress, significant (*P*< 0·01) correlations were detected between grain yield and all measured traits except husk cover. Husk cover had no significant (*P*< 0·05) correlation with any of the measured traits, except plant height and ears per plant. Of the 11 traits measured, only stay green characteristic had significant (*P*< 0·01) correlations with all other traits (Table 3). Under rain-fed conditions, grain yield of the extra-early maturing maize hybrids showed positive and significant (P< 0·01) correlations with plant height, ear height and ears per plant. Significant (*P*< 0·01) and negative correlations were detected between grain yield and days to 50% anthesis, days to 50% silking, anthesis and silking interval, plant aspect and ear aspect (Table 3). The flowering traits had significant (*P*< 0·01) correlations with all the other agronomic traits except for plant height and husk cover (Table 3).

**Table 3:** Correlation coefficients between grain yield and other agronomic traits of extra-early maize hybrids under drought (above diagonal) and under rainfed conditions (below diagonal) in Nigeria during the 2010 and 2011 growing seasons

	Grain yield	DA	DS	ASI	PLHT	EHT	PASP	EASP	EPP	HUSK	SGC
Grain yield		−0·59(*P* < 0·01)	−0·70(*P* < 0·01)	−0·59(*P* < 0·01)	0·64(*P* < 0·01)	0·69(*P* < 0·01)	−0·83(*P* < 0·01)	−0·86(*P* < 0·01)	0·72(*P* < 0·01)	−0·04(NS)	−0·53(*P* < 0·01)
DA	−0·11(*P* < 0·01)		0·84(*P* < 0·01)	0·40(*P* < 0·01)	−0·30(*P* < 0·01)	−0·35(*P* < 0·01)	0·45(*P* < 0·01)	0·45(*P* < 0·01)	−0·53(*P* < 0·01)	−0·06(NS)	0·25(*P* < 0·01)
DS	−0·20(*P* < 0·01)	0·91(*P* < 0·01)		0·83(*P* < 0·01)	−0·40(*P* < 0·01)	−0·49(*P* < 0·01)	0·62(*P* < 0·01)	0·63(*P* < 0·01)	−0·66(*P* < 0·01)	0·01(NS)	0·42(*P* < 0·01)
ASI	−0·26(*P* < 0·01)	0·20(*P* < 0·01)	0·57(*P* < 0·01)		−0·37(*P* < 0·01)	−0·46(*P* < 0·01)	0·59(*P* < 0·01)	0·65(*P* < 0·01)	−0·58(*P* < 0·01)	0·08(NS)	0·47(*P* < 0·01)
PLHT	0·63(*P* < 0·01)	−0·02(NS)	−0·06(NS)	−0·02(NS)		0·80(*P* < 0·01)	−0·74(*P* < 0·01)	−0·61(*P* < 0·01)	0·48(*P* < 0·01)	−0·19(*P* < 0·05)	−0·39(*P* < 0·01)
EHT	0·43(*P* < 0·01)	−0·17(*P* < 0·01)	−0·21(*P* < 0·05)	−0·18(*P* < 0·05)	0·68(*P* < 0·01)		−0·75(*P* < 0·01)	−0·67(*P* < 0·01)	0·50(*P* < 0·01)	0·06(NS)	−0·28(*P* < 0·01)
PASP	−0·62(*P* < 0·01)	0·18(*P* < 0·01)	0·25(*P* < 0·01)	0·23(*P* < 0·01)	−0·56(*P* < 0·01)	−0·52(*P* < 0·01)		0·81(*P* < 0·01)	−0·70(*P* < 0·01)	0·15(NS)	0·60(*P* < 0·01)
EASP	−0·67(*P* < 0·01)	0·13(*P* < 0·01)	0·21(*P* < 0·01)	0·27(*P* < 0·01)	−0·49(*P* < 0·01)	−0·49(*P* < 0·01)	0·57(*P* < 0·01)		−0·67(*P* < 0·01)	0·13(NS)	0·49(*P* < 0·01)
EPP	0·52(*P* < 0·01)	−0·09(*P* < 0·01)	−0·15(*P* < 0·01)	−0·19(*P* < 0·01)	0·26(*P* < 0·01)	0·22(*P* < 0·01)	−0·30(*P* < 0·01)	−0·38(*P* < 0·01)		−0·21(*P* < 0·05)	−0·42(*P* < 0·01)
HUSK	0·05(NS)	0·10(*P* < 0·01)	−0·07(NS)	−0·05(NS)	−0·02(NS)	0·10(*P* < 0·01)	0·08(*P* < 0·01)	−0·08(*P* < 0·05)	0·06(NS)		0·22(*P* < 0·01)

DA, days to anthesis; DS, days to silking; ASI, anthesis-silking interval; PLHT, plant height; EHT, ear height; HC, husk cover; PASP, plant aspect; EASP, ear aspect; EPP, ears per plant; SGC, stay green characteristic; NS, not significant.

**Table 4:** Means squares from line × tester analysis for grain yield and other agronomic traits of 120 extra-early maturing maize hybrids evaluated under managed drought at Ikenne during 2010/11 and 2011/12 dry seasons, terminal drought at Bagauda in 2010 and rain fed conditions at Ikenne in 2010 and 2011 rainy season and at Bagauda during 2011 rainy season

Source	DF	Grainyield (kg/ha)	DA	DS	ASI (days)	Plantheight (cm)	Earheight (cm)	Plant aspect(scale 1–5)	Ear aspect(scale 1–5)	Earper plant	SGC
Drought											
REP	1	549 473·4 (NS)	17 (*P* < 0·01)	34 (*P* < 0·05)	2 (NS)	4693 (*P* < 0·01)	2720 (*P* < 0·01)	0·2 (NS)	0·1 (NS)	0·01 (NS)	14·6 (*P* < 0·01)
E	2	323 675 119·2 (*P* < 0·01)	1297 (*P* < 0·01)	101 (*P* < 0·01)	1282 (*P* < 0·01)	69 857 (*P* < 0·01)	2914 (*P* < 0·01)	16·6 (*P* < 0·01)	36·5 (*P* < 0·01)	12·57 (*P* < 0·01)	145·7 (*P* < 0·01)
Block(E × REP)	60	272 586 (*P* < 0·01)	5 (*P* < 0·01)	15 (*P* < 0·01)	9 (*P* < 0·01)	673 (*P* < 0·01)	308 (*P* < 0·01)	0·5 (*P* < 0·01)	0·3 (*P* < 0·01)	0·03 (*P* < 0·01)	1·0 (*P* < 0·01)
REP(E)	3	421 600 (*P* < 0·05)	9 (*P* < 0·01)	13 (NS)	4 (NS)	3404 (*P* < 0·01)	2050 (*P* < 0·01)	0·2 (NS)	0·8 (*P* < 0·01)	0·06 (*P* < 0·05)	10·1 (*P* < 0·01)
Hybrid	119	839 424 (*P* < 0·01)	9 (*P* < 0·01)	26 (*P* < 0·01)	9 (*P* < 0·01)	495 (*P* < 0·01)	284 (*P* < 0·01)	0·5 (*P* < 0·01)	0·6 (*P* < 0·01)	0·04 (*P* < 0·01)	0·7 (*P* < 0·01)
^GCA^LINE	40	416 836·2 (*P* < 0·01)	10 (*P* < 0·01)	24 (*P* < 0·01)	10 (*P* < 0·01)	477 (*P* < 0·01)	317 (*P* < 0·01)	0·3 (*P* < 0·01)	0·4 (*P* < 0·01)	0·04 (*P* < 0·01)	0·9 (*P* < 0·01)
^GCA^TESTER	2	23 730 750·9 (*P* < 0·01)	154 (*P* < 0·01)	568 (*P* < 0·01)	150 (*P* < 0·01)	9257 (*P* < 0·01)	6433 (*P* < 0·01)	12·9 (*P* < 0·01)	11·5 (*P* < 0·01)	0·46 (*P* < 0·01)	5·5 (*P* < 0·01)
E × GCA_LINE_	80	356 506·2 (*P* < 0·01)	5 (*P* < 0·01)	15 (*P* < 0·01)	8 (*P* < 0·01)	382 (*P* < 0·01)	172 (*P* < 0·01)	0·3 (*P* < 0·05)	0·3 (*P* < 0·01)	0·03 (*P* < 0·05)	0·9 (*P* < 0·01)
^E × GCA^TESTER	4	4 483 597·9 (*P* < 0·01)	10 (*P* < 0·01)	42 (*P* < 0·01)	43 (*P* < 0·01)	364 ns	416 (*P* < 0·01)	1·4 (*P* < 0·01)	1·3 (*P* < 0·01)	0·04 (NS)	5·1 (*P* < 0·01)
^SCA^LINE × TESTER	77	624 851·3 (*P* < 0·01)	6 (*P* < 0·01)	14 (*P* < 0·01)	6 (NS)	393 (NS)	165 (*P* < 0·01)	0·4 (*P* < 0·01)	0·4 (*P* < 0·01)	0·04 (*P* < 0·01)	0·7 (*P* < 0·05)
E× ^SCA^LINE × TESTER	150	280 846·1 (*P* < 0·01)	3 (*P* < 0·01)	8 (NS)	5 (NS)	381 (*P* < 0·01)	120 (NS)	0·3 (*P* < 0·05)	0·2 (NS)	0·02 (NS)	0·7 (*P* < 0·05)
Error	358	162 414	2·1	8·7	5·6	261·4	107·0	0·20	0·19	0·020	0·55
Rain-fed condition											
Rep	1	42 144 825 (*P* < 0·05)	1 (NS)	0 (NS)	0·6 (NS)	392 (NS)	2 (NS)	2 (NS)	7 (NS)	0·02 (*P* < 0·05)	
E	2	559 444 285 (*P* < 0·01)	304 (*P* < 0·01)	315 (*P* < 0·01)	0·3 (NS)	9523 (*P* < 0·01)	179 (NS)	7 (*P* < 0·05)	22 (*P* < 0·01)	0·32 (*P* < 0·01)	
Blk (E × Rep)	60	6 039 457 (NS)	3 (*P* < 0·01)	4 (*P* < 0·01)	0·7 (*P* < 0·05)	327 (*P* < 0·01)	200 (*P* < 0·01)	1 (NS)	4 (*P* < 0·01)	0·00 (NS)	
Rep (E)	3	17 164 104 (*P* < 0·05)	1 (NS)	1 (NS)	1·4 (*P* < 0·05)	1748 (*P* < 0·01)	773 (*P* < 0·01)	2 (NS)	10 (*P* < 0·01)	0·02 (*P* < 0·05)	
Hybrid	119	7 897 078 (*P* < 0·05)	6 (*P* < 0·01)	10 (*P* < 0·01)	1·5 (*P* < 0·01)	461 (*P* < 0·01)	367 (*P* < 0·01)	2 (*P* < 0·01)	4 (*P* < 0·01)	0·01 (*P* < 0·01)	
GCALine	40	7 665 893 (*P* < 0·05)	9 (*P* < 0·01)	14 (*P* < 0·01)	2·1 (*P* < 0·01)	444 (*P* < 0·01)	435 (*P* < 0·01)	2 (NS)	4 (*P* < 0·05)	0·01 (*P* < 0·01)	
GCATester	2	1 052 887 (*P* < 0·01)	75·28 (*P* < 0·01)	141 (*P* < 0·01)	11 (*P* < 0·01)	13 293 (*P* < 0·01)	10 047 (*P* < 0·01)	16 (*P* < 0·01)	4 (NS)	0·11 (*P* < 0·01)	
E × GCALine	80	6 540 379 (*P* < 0·05)	2 (*P* < 0·01)	3 (*P* < 0·01)	0·7 (NS)	186 (*P* < 0·05)	166 (*P* < 0·01)	2 (NS)	5 (*P* < 0·01)	0·01 (*P* < 0·01)	
E × GCATester	4	59 025 730 (*P* < 0·01)	4 (*P* < 0·01)	13 (*P* < 0·01)	4·9 (*P* < 0·01)	488 (*P* < 0·01)	823 (*P* < 0·01)	5 (*P* < 0·05)	20 (*P* < 0·01)	0·02 (*P* < 0·05)	
SCA Line × Tester	77	8 550 463 (*P* < 0·05)	5 (*P* < 0·01)	8 (*P* < 0·01)	1·2 (*P* < 0·01)	262 (*P* < 0·01)	177 (*P* < 0·01)	2 (*P* < 0·05)	5 (*P* < 0·01)	0·01 (*P* < 0·01)	
E × SCALine × Tester	152	6 768 910 (*P* < 0·05)	2 (*P* < 0·01)	3 (*P* < 0·01)	0·8 (*P* < 0·01)	190 (*P* < 0·01)	137 (*P* < 0·01)	2 (NS)	6 (*P* < 0·01)	0·01 (*P* < 0·05)	
Error	361	6 608 510	1·3	1·9	0·52	130·6	93·6	1·6	2·6	0·010	

DF, degrees of freedom; DA, days to anthesis; DYSK, days to silk; ASI, anthesis-silking interval; SGC, stay green characteristic; E, environment; Rep, replication; Blk, block; NS,not significant.

General and specific combining abilities of grain yield and other traits of extra-early maturing inbreds under drought and rain-fed conditions

Partitioning of the hybrids into GCA and SCA components revealed that GCA_line_, GCA_tester_ and SCA_line × tester_ mean squares were significant (*P* < 0·01) for all traits measured under the two different research conditions (Table 4). Under drought, the mean squares for environment (E) × GCA_line_ and E×GCA_tester_ interactions were significant (*P* < 0·01) for yield and all measured traits except the E×GCA_tester_ for plant height and ears per plant. However, the E × SCA_line × tester_ interaction means squares were not significant (*P*< 0·05) for days to silking, anthesis-silking interval, ear height, ear aspect and ears per plant under drought. Similarly, the E × GCA_line_, E × GCA_tester_ and E × SCA_line × tester_ mean squares were significant (*P*< 0·05) for grain yield and most measured traits except for E × GCA_line_ for anthesis-silking interval and plant aspect and E × SCA_line × tester_ for plant aspect under rain-fed conditions. The GCA effects accounted for 72% of the total variation for grain yield under drought (Table 5). In contrast, the SCA effects accounted for 52% of the total variation for grain yield under rainfed conditions (Table 6). Across the research conditions, the proportions of the GCA effects for yield and all measured traits were higher than the SCA effects except for grain yield and ear aspect under rain-fed conditions. Under drought, significant (*P*< 0·05) for grain yield and most measured traits except for E × GCA_line_ for anthesis-silking interval and plant aspect and E × SCA_line × tester_ for plant aspect under rain-fed conditions. The GCA effects accounted for 72% of the total variation for grain yield under drought (Table 5). In contrast, the SCA effects accounted for 52% of the total variation for grain yield under rainfed conditions (Table 6). Across the research conditions, the proportions of the GCA effects for yield and all measured traits were higher than the SCA effects except for grain yield and ear aspect under rain-fed conditions.

**Table 5:** General combining ability effects of extra-early yellow inbred parents for grain yield and other agronomic traits evaluated under induced drought at Ikenne during the dry seasons of 2010/11 and 2011/12 and terminal drought at Bagauda in 2010

INBREDS	Pedigree	YIELD	POLLEN	DYSK	ASI	PLHT	EHT	PASP	EASP	EPP	SGC
TZEEI 9	TZEF-Y SR BC1 × 9450 STR S6 Inb 8A	169 (NS)	−1·8 (*P*< 0·01)	−1·9 (*P*< 0·05)	0·0 (NS)	−3·1 (NS)	−1·4 (NS)	−0·1 (NS)	−0·1 (NS)	0·1 (NS)	−0·0 (NS)
TZEEI 58	TZEE-Y SR BC1 × 9450 STR S6 Inb 1A	−149 (NS)	0·4 (NS)	1·2 (NS)	0·8 (NS)	5·6 (NS)	2·4 (NS)	0·0 (NS)	0·1 (NS)	−0·0 (NS)	−0·1 (NS)
TZEEI 59	TZEE-Y SR BC1 × 9450 STR S6 Inb 3A	−52 (NS)	−0·5 (NS)	−0·3 (NS)	0·2 (NS)	−0·5 (NS)	0·7 (NS)	0·1 (NS)	0·0 (NS)	0·0 (NS)	0·4 (NS)
TZEEI 60	TZEE-Y SR BC1 × 9450 STR S6 Inb 3B	−85 (NS)	−1·1 (*P*< 0·05)	−1·7 (NS)	−0·6 (NS)	−1·2 (NS)	−3·7 (NS)	−0·1 (NS)	0·1 (NS)	0·0 (NS)	−0·1 (NS)
TZEEI 61	TZEE-Y SR BC1 × 9450 STR S6 Inb 4B	79 (NS)	0·7 (NS)	1·7 (NS)	1·0 (NS)	0·3 (NS)	−1·7 (NS)	0·1 (NS)	−0·1 (NS)	−0·0 (NS)	0·2 (NS)
TZEEI 62	TZEE-Y SR BC1 × 9450 STR S6 Inb 7A	−251 (NS)	0·6 (NS)	3·2 (*P*< 0·01)	2·9 (*P*< 0·01)	−5·1 (NS)	0·1 (NS)	0·3 (*P*< 0·05)	0·4 (*P*< 0·01)	−0·1 (*P*< 0·05)	0·6 (*P*< 0·01)
TZEEI 63*	TZEE-Y SR BC1 × 9450 STR S6 Inb 7B	−379 (*P*< 0·01)	0·8 (*P*< 0·01)	1·8 (*P*< 0·01)	0·9 (*P*< 0·05)	−5·2 (*P*< 0·01)	−5·4 (*P*< 0·01)	0·3 (*P*< 0·01)	0·3 (*P*< 0·01)	−0·1 (*P*< 0·01)	0·2 (NS)
TZEEI 64	TZEE-Y SR BC1 × 9450 STR S6 Inb 8A	−26 (NS)	−1·0 (*P*< 0·05)	−1·2 (NS)	−0·3 (NS)	0·2 (NS)	−0·4 (NS)	0·0 (NS)	0·0 (NS)	0·0 (NS)	−0·0 (NS)
TZEEI 65	TZEE-Y SR BC1 × 9450 STR S6 Inb 8C	−188 (NS)	0·8 (NS)	1·5 (NS)	0·7 (NS)	2·8 (NS)	−3·8 (NS)	−0·0 (NS)	0·0 (NS)	0·0 (NS)	−0·3 (NS)
TZEEI 66	TZEE-Y SR BC1 × 9450 STR S6 Inb 9A	−192 (NS)	0·6 (NS)	0·5 (NS)	−0·2 (NS)	−0·4 (NS)	−1·9 (NS)	0·0 (NS)	0·0 (NS)	−0·1 (NS)	0·2 (NS)
TZEEI 67	TZEE-Y SR BC1 × 9450 STR S6 Inb 10B	254 (NS)	0·3 (NS)	−0·3 (NS)	−0·6 (NS)	4·4 (NS)	5·4 (NS)	−0·2 (NS)	−0·4 (*P*< 0·01)	0·0 (NS)	0·1 (NS)
TZEEI 68	TZEE-Y SR BC1 × 9450 STR S6 Inb 11	−144 (NS)	−0·1 (NS)	−0·8 (NS)	−0·7 (NS)	−8·4 (NS)	−5·8 (NS)	0·2 (NS)	0·3 (*P*< 0·05)	0·0 (NS)	−0·1 (NS)
TZEEI 69	TZEE-Y SR BC1 × 9450 STR S6 Inb 34-2-2	83 (NS)	−1·2 (*P*< 0·05)	−1·9 (*P*< 0·05)	−0·7 (NS)	4·0 (NS)	3·3 (NS)	0·1 (NS)	0·1 (NS)	0·0 (NS)	0·1 (NS)
TZEEI 70	TZEF-Y SR BC1 × 9450 STR S6 Inb 1A	16 (NS)	0·4 (NS)	0·8 (NS)	0·4 (NS)	3·9 (NS)	0·3 (NS)	−0·1 (NS)	−0·1 (NS)	0·0 (NS)	−0·4 (NS)
TZEEI 71	TZEF-Y SR BC1 × 9450 STR S6 Inb 2B	141 (NS)	−0·2 (NS)	−0·4 (NS)	−0·3 (NS)	4·7 (NS)	2·0 (NS)	−0·1 (NS)	−0·0 (NS)	0·0 (NS)	−0·3 (NS)
TZEEI 72	TZEF-Y SR BC1 × 9450 STR S6 Inb 2C	−171 (NS)	−0·2 (NS)	−0·2 (NS)	−0·0 (NS)	−4·6 (NS)	−3·0 (NS)	0·1 (NS)	0·2 (NS)	0·1 (NS)	−0·1 (NS)
TZEEI 73	TZEF-Y SR BC1 × 9450 STR S6 Inb 3A	55 (NS)	−0·5 (NS)	−0·1 (NS)	0·4 (NS)	1·5 (NS)	−0·3 (NS)	−0·2 (NS)	0·0 (NS)	0·1 (*P*< 0·05)	0·2 (NS)
TZEEI 74	TZEF-Y SR BC1 × 9450 STR S6 Inb 5A	−171 (NS)	−0·6 (NS)	−0·4 (NS)	0·2 (NS)	−6·9 (NS)	−7·0 (*P*< 0·05)	0·2 (NS)	0·2 (NS)	−0·0 (NS)	0·2 (NS)
TZEEI 75	TZEF-Y SR BC1 × 9450 STR S6 Inb 7B	−222 (NS)	0·8 (NS)	1·9 (*P*< 0·05)	1·0 (NS)	−8·6 (NS)	−10·5 (*P*< 0·01)	0·4 (*P*< 0·01)	0·3 (*P*< 0·05)	−0·1 (*P*< 0·01)	0·1 (NS)
TZEEI 76	TZEF-Y SR BC1 × 9450 STR S6 Inb 8B	−53 (NS)	0·1 (NS)	0·4 (NS)	0·2 (NS)	1·4 (NS)	−3·0 (NS)	0·1 (NS)	0·1 (NS)	0·0 (NS)	−0·3 (NS)
TZEEI 77	TZEE-Y Pop Co S6 Inbred 34	−280 (*P*< 0·05)	−1·4 (*P*< 0·01)	−1·3 (NS)	0·0 (NS)	−12·9 (*P*< 0·01)	−6·0 (*P*< 0·05)	0·2 (NS)	0·2 (NS)	−0·1 (NS)	0·4 (NS)
TZEEI 78	TZEE-Y Pop Co S6 Inbred 44	−110 (NS)	0·8 (NS)	1·4 (NS)	0·5 (NS)	3·0 (NS)	1·5 (NS)	−0·0 (NS)	0·1 (NS)	−0·1 (*P*< 0·05)	0·0 (NS)
TZEEI 79*	TZEEY Pop Co S6 Inbred 47-2-4A	199 (NS)	−0·4 (NS)	−1·0 (*P*< 0·05)	−0·7 (NS)	7·1 (*P*< 0·01)	5·0 (*P*< 0·01)	−0·2 (*P*< 0·01)	−0·2 (*P*< 0·05)	0·0 (*P*< 0·01)	−0·1 (NS)
TZEEI 80	TZEF-Y SR BC1 × 9450 STR S6 Inb 8C	58 (NS)	−0·2 (NS)	−0·5 (NS)	−0·3 (NS)	6·2 (NS)	0·6 (NS)	−0·1 (NS)	−0·0 (NS)	0·0 (NS)	−0·4 (NS)
TZEEI 81	TZEF-Y SR BC1 × 9450 STR S6 Inb 9A	248 (NS)	0·5 (NS)	1·0 (NS)	0·5 (NS)	−0·5 (NS)	0·4 (NS)	0·1 (NS)	−0·1 (NS)	−0·1 (NS)	−0·0 (NS)
TZEEI 82	TZEF-Y SR BC1 × 9450 STR S6 Inb 10B	112 (NS)	−0·4 (NS)	−0·3 (NS)	0·1 (NS)	9·2 (*P*< 0·05)	5·5 (NS)	−0·2 (NS)	−0·2 (NS)	0·0 (NS)	−0·2 (NS)
TZEEI 83	TZEF-Y SR BC1 × 9450 STR S6 Inb 10C	46 (NS)	−1·4 (*P*< 0·01)	−1·6 (NS)	−0·3 (NS)	−0·7 (NS)	2·2 (NS)	0·0 (NS)	0·1 (NS)	0·0 (NS)	0·2 (NS)
TZEEI 86	TZEF-Y POP STR COS6 Inb 47-2-4A	135 (NS)	−0·4 (NS)	0·3 (NS)	0·6 (NS)	−4·8 (NS)	−0·1 (NS)	0·0 (NS)	−0·2 (NS)	0·0 (NS)	−0·1 (NS)
TZEEI 87	TZEF-Y POP STR COS6 Inb 47-24B	−53 (NS)	0·2 (NS)	−0·7 (NS)	−0·9 (NS)	−1·2 (NS)	3·5 (NS)	−0·0 (NS)	−0·2 (NS)	−0·0 (NS)	−0·1 (NS)
TZEEI 88	TZEF-YSR BC1 X 9450 STR S6inb 42-2-2	40 (NS)	1·6 (*P*< 0·01)	1·7 (NS)	0·1 (NS)	−3·4 (NS)	2·4 (NS)	0·0 (NS)	−0·1 (NS)	−0·0 (NS)	0·3 (NS)
TZEEI 89	TZEF-YSR BC1 X 9450 STR S6inb 13A	−36 (NS)	0·3 (NS)	−0·7 (NS)	−1·0 (NS)	−4·8 (NS)	−3·0 (*P*< 0·05)	0·2 (NS)	−0·0 (NS)	−0·0 (NS)	0·3 (NS)
TZEEI 94	TZEE-Y Pop Co S6 Inbred 47-2-4B	225 (NS)	0·1 (NS)	−0·7 (NS)	−0·9 (NS)	2·6 (NS)	6·9 (*P*< 0·05)	−0·2 (NS)	−0·2 (NS)	0·1 (*P*< 0·05)	−0·0 (NS)
TZEEI 95	TZEE-Y Pop Co S6 Inbred 47-3-4	178 (NS)	−0·5 (*P*< 0·05)	−0·7 (NS)	−0·2 (NS)	−2·1 (NS)	0·3 (NS)	−0·0 (NS)	−0·1 (NS)	0·0 (NS)	−0·1 (NS)
TZEEI 96	TZEE-Y Pop Co S6 Inbred 78	−8 (NS)	0·7 (NS)	1·3 (NS)	0·6 (NS)	3·4 (NS)	4·1 (NS)	−0·1 (NS)	0·1 (NS)	−0·0 (NS)	−0·3 (NS)
TZEEI 97	TZEE-Y Pop Co S6 Inbred 101-1-2	308 (*P*< 0·05)	−1·0 (*P*< 0·05)	−1·5 (NS)	−0·5 (NS)	8·5 (NS)	6·2 (*P*< 0·05)	−0·1 (NS)	−0·2 (NS)	0·1 (*P*< 0·05)	−0·2 (NS)
TZEEI 98	TZEE-Y Pop Co S6 Inbred 101-2-4	223 (NS)	0·3 (NS)	−0·9 (NS)	−1·1 (NS)	1·7 (NS)	2·0 (NS)	−0·1 (NS)	−0·3 (*P*< 0·05)	0·1 (*P*< 0·05)	−0·3 (NS)
TZEEI 99	TZEE-Y Pop Co S6 Inbred 101-1-4	−4 (NS)	1·2 (*P*< 0·05)	0·8 (NS)	−0·4 (NS)	4·0 (NS)	3·8 (NS)	0·1 (NS)	0·0 (NS)	−0·0 (NS)	0·1 (NS)
TZEEI 100	TZEF-Y POP STR COS6 inb 47-3-4	114 (NS)	−0·2 (NS)	−1·3 (NS)	−1·2 (NS)	2·8 (NS)	8·3 (*P*< 0·01)	−0·2 (*P*< 0·05)	0·0 (NS)	0·0 (NS)	0·1 (NS)
TZEEI 101	TZEF-Y POP STR COS6 inb 101-1-4	135 (NS)	0·3 (NS)	0·6 (NS)	0·4 (NS)	2·9 (NS)	2·6 (NS)	−0·1 (NS)	−0·2 (NS)	−0·1 (NS)	0·0 (NS)
TZEEI 102	TZEF-Y SR BC1 X 9450 STRS6 inb 7A	−159 (NS)	0·5 (NS)	0·7 (NS)	0·1 (NS)	−6·4 (NS)	−8·4 (*P* < 0·01)	0·1 (NS)	0·2 (NS)	−0·0 (NS)	−0·3 (NS)
TZEEI 108	TZEF-Y SR BC1 X 9450 STR inb 7-1-2A	−5 (NS)	0·6 (NS)	1·3 (NS)	0·6 (NS)	8·9 (*P* < 0·05)	3·3 (NS)	−0·1 (NS)	−0·1 (NS)	−0·0 (NS)	0·1 (NS)
TZEEI 115	TZEF-Y SR BC1 X 9450 STR inb 5	−96 (NS)	0·3 (NS)	−0·1 (NS)	−0·4 (NS)	−7·3 (NS)	−7·2 (*P* < 0·05)	0·1 (NS)	0·1 (NS)	0·0 (NS)	0·1 (NS)
S.E.		139·0 (NS)	0·5 (NS)	0·9 (NS)	0·7 (NS)	4·6 (NS)	3·1 (NS)	0·1 (NS)	0·1 (NS)	0·0 (NS)	0·2 (NS)
Proportion of GCA over SCA		0·72	0·75	0·99	0·76	0·71	0·80	0·72	0·75	0·65	0·62

DYSK, days to silk; ASI, anthesis-silking interval; PLHT, plant height; EHT, ear height; PASP, plant aspect; EASP, ear aspect; EPP, ears per plant; SGC, stay green characteristic; NS, not significant; GCA, general combining ability; SCA, specific combining ability; S.E., standard error between two GCA effects.

**Table 6:** General combining ability effects of extra-early yellow inbred parents for grain yield and other agronomic traits evaluated under rain-fed conditions at Ikenne and Bagauda in 2010 and 2011

INBREDS	Pedigree	YIELD	POLLEN	DYSK	ASI	PLHT	EHT	PASP	HC	EASP	EPP
TZEEI 9	TZEF-Y SR BC1 × 9450 STR S6 Inb 8A	−480 (NS)	−0·5 (NS)	−0·9 (*P*< 0·05)	−0·4 (*P*< 0·05)	−4 (NS)	−4 (NS)	0·2 (NS)	−0·1 (NS)	0·5 (NS)	−0·0 (NS)
TZEEI 58	TZEE-Y SR BC1 × 9450 STR S6 Inb 1A	−345 (NS)	0·3 (NS)	0·4 (NS)	0·1 (NS)	11 (*P*< 0·01)	3 (NS)	−0·3 (NS)	−0·2 (NS)	−0·2 (NS)	0·0 (NS)
TZEEI 59	TZEE-Y SR BC1 × 9450 STR S6 Inb 3A	−235 (NS)	−0·3 (NS)	−0·1 (NS)	0·2 (NS)	−2 (NS)	2 (NS)	0·1 (NS)	0·3 (*P*< 0·01)	0·0 (NS)	−0·0 (NS)
TZEEI 60	TZEE-Y SR BC1 × 9450 STR S6 Inb 3B	124 (NS)	−0·6 (NS)	−0·9 (*P*< 0·05)	−0·3 (NS)	−4 (NS)	−5 (NS)	0·1 (NS)	−0·0 (NS)	−0·5 (NS)	0·0 (NS)
TZEEI 61	TZEE-Y SR BC1 × 9450 STR S6 Inb 4B	444 (NS)	0·2 (NS)	0·4 (NS)	0·2 (NS)	2 (NS)	−2 (NS)	−0·2 (NS)	−0·0 (NS)	−0·2 (NS)	0·0 (NS)
TZEEI 62	TZEE-Y SR BC1 × 9450 STR S6 Inb 7A	−434 (NS)	0·8 (NS)	1·3 (*P*< 0·01)	0·4 (*P*< 0·05)	−1 (NS)	−0 (NS)	−0·1 (NS)	−0·1 (NS)	−0·9 (NS)	−0·1 (*P*< 0·01)
TZEEI 63*	TZEE-Y SR BC1 × 9450 STR S6 Inb 7B	−2 (NS)	0·6 (*P*< 0·01)	0·8 (*P*< 0·01)	0·2 (NS)	−2 (NS)	−6 (NS)	0·1 (NS)	−0·2 (*P*< 0·05)	−0·0 (NS)	−0·0 (*P*< 0·01)
TZEEI 64	TZEE-Y SR BC1 × 9450 STR S6 Inb 8A	−184 (NS)	−0·9 (*P*< 0·01)	−1·2 (*P*< 0·01)	−0·3 (NS)	−3 (NS)	−2 (NS)	0·0 (NS)	0·0 (NS)	0·1 (NS)	0·0 (NS)
TZEEI 65	TZEE-Y SR BC1 × 9450 STR S6 Inb 8C	−530 (NS)	1·0 (*P*< 0·01)	1·2 (*P*< 0·01)	0·2 (NS)	−4 (NS)	−7 (*P*< 0·05)	0·1 (NS)	−0·0 (NS)	0·0 (NS)	−0·0 (NS)
TZEEI 66	TZEE-Y SR BC1 × 9450 STR S6 Inb 9A	−177 (NS)	0·4 (NS)	0·7 (NS)	0·6 (*P*< 0·01)	−1 (NS)	1 (NS)	−0·1 (NS)	−0·0 (NS)	0·5 (NS)	0·0 (NS)
TZEEI 67	TZEE-Y SR BC1 × 9450 STR S6 Inb 10B	275 (NS)	0·6 (NS)	0·0 (NS)	−0·6 (*P*< 0·01)	4 (NS)	10 (*P*< 0·01)	−0·5 (NS)	0·3 (*P*< 0·01)	−0·3 (NS)	0·0 (NS)
TZEEI 68	TZEE-Y SR BC1 × 9450 STR S6 Inb 11	−173 (NS)	−0·3 (NS)	−0·6 (NS)	−0·3 (NS)	−5 (NS)	−2 (NS)	0·1 (NS)	0·2 (*P*< 0·05)	0·5 (NS)	0·0 (NS)
TZEEI 69	TZEE-Y SR BC1 × 9450 STR S6 Inb 34-2-2	−53 (NS)	−0·8 (*P*< 0·05)	−0·8 (NS)	−0·0 (NS)	3 (NS)	3 (NS)	0·1 (NS)	0·1 (NS)	−0·5 (NS)	0·0 (NS)
TZEEI 70	TZEF-Y SR BC1 × 9450 STR S6 Inb 1A	−1484 (NS)	−0·3 (NS)	−0·6 (NS)	−0·3 (NS)	3 (NS)	4 (NS)	−0·2 (NS)	0·1 (NS)	−0·8 (NS)	0·1 (*P*< 0·01)
TZEEI 71	TZEF-Y SR BC1 × 9450 STR S6 Inb 2B	94 (NS)	−0·3 (NS)	−0·4 (NS)	−0·2 (NS)	−2 (NS)	−2 (NS)	−0·0 (NS)	−0·1 (NS)	−1·3 (*P*< 0·01)	−0·0 (*P*< 0·05)
TZEEI 72	TZEF-Y SR BC1 × 9450 STR S6 Inb 2C	−151 (NS)	0·1 (NS)	0·1 (NS)	−0·0 (NS)	−4 (NS)	−3 (NS)	0·2 (NS)	−0·1 (NS)	−0·6 (NS)	0·0 (NS)
TZEEI 73	TZEF-Y SR BC1 × 9450 STR S6 Inb 3A	252 (NS)	−0·3 (NS)	0·1 (NS)	0·4 (*P*< 0·05)	5 (NS)	2 (NS)	−0·3 (NS)	−0·3 (*P*< 0·01)	0·5 (NS)	0·0 (NS)
TZEEI 74	TZEF-Y SR BC1 × 9450 STR S6 Inb 5A	−612 (NS)	−0·8 (*P*< 0·05)	−0·9 (*P*< 0·05)	−0·2 (NS)	−2 (NS)	−3 (NS)	0·2 (NS)	−0·1 (NS)	0·5 (NS)	−0·0 (NS)
TZEEI 75	TZEF-Y SR BC1 × 9450 STR S6 Inb 7B	−348 (NS)	0·3 (NS)	0·9 (*P*< 0·05)	0·6 (*P*< 0·01)	−11 (*P*< 0·01)	−14 (*P*< 0·01)	0·1 (NS)	−0·0 (NS)	−0·2 (NS)	−0·0 (NS)
TZEEI 76	TZEF-Y SR BC1 × 9450 STR S6 Inb 8B	−179 (NS)	−0·5 (NS)	−0·5 (NS)	0·0 (NS)	−1 (NS)	−1 (NS)	−0·2 (NS)	0·0 (NS)	0·1 (NS)	0·0 (NS)
TZEEI 77	TZEE-Y Pop Co S6 Inbred 34	−252 (NS)	−1·7 (*P*< 0·01)	−2·0 (*P*< 0·01)	−0·4 (NS)	−8 (*P*< 0·05)	−5 (NS)	0·2 (NS)	−0·0 (NS)	−0·1 (NS)	−0·0 (NS)
TZEEI 78	TZEE-Y Pop Co S6 Inbred 44	−471 (NS)	1·1 (*P*< 0·01)	2·0 (*P*< 0·01)	0·9 (*P*< 0·01)	6 (NS)	3 (NS)	−0·0 (NS)	−0·3 (*P*< 0·01)	−0·2 (NS)	−0·0 (NS)
TZEEI 79*	TZEE-Y Pop Co S6 Inbred 47-2-4A	101 (NS)	−0·2 (NS)	−0·1 (NS)	−0·0 (NS)	8 (*P*< 0·01)	7 (*P*< 0·01)	−0·3 (*P*< 0·05)	0·0 (NS)	0·1 (NS)	0·0 (NS)
TZEEI 80	TZEF-Y SR BC1 × 9450 STR S6 Inb 8C	220 (NS)	−0·3 (NS)	−0·4 (NS)	−0·1 (NS)	−3 (NS)	−6 (*P*< 0·05)	0·1 (NS)	0·1 (NS)	0·7 (NS)	0·0 (NS)
TZEEI 81	TZEF-Y SR BC1 × 9450 STR S6 Inb 9A	395 (NS)	−0·1 (NS)	−0·4 (NS)	−0·4 (NS)	7 (*P*< 0·05)	7 (*P*< 0·05)	−0·4 (NS)	−0·0 (NS)	0·2 (NS)	0·0 (NS)
TZEEI 82	TZEF-Y SR BC1 × 9450 STR S6 Inb 10B	76 (NS)	0·3 (NS)	0·9 (*P*< 0·05)	0·6 (*P*< 0·01)	0 (NS)	1 (NS)	0·1 (NS)	−0·1 (NS)	0·6 (NS)	−0·0 (NS)
TZEEI 83	TZEF-Y SR BC1 × 9450 STR S6 Inb 10C	−165 (NS)	−0·6 (NS)	−0·9 (*P*< 0·05)	−0·4 (NS)	3 (NS)	2 (NS)	−0·1 (NS)	0·1 (NS)	0·3 (NS)	0·0 (NS)
TZEEI 86	TZEF-Y POP STR COS6 Inb 47-2-4A	433 (NS)	−1·0 (*P*< 0·01)	−1·3 (*P*< 0·01)	−0·2 (NS)	1 (NS)	−3 (NS)	−0·1 (NS)	0·1 (NS)	−0·8 (NS)	0·0 (NS)
TZEEI 87	TZEF-Y POP STR COS6 Inb 47-24B	73 (NS)	0·8 (*P*< 0·05)	0·5 (NS)	0·0 (NS)	−2 (NS)	4 (NS)	−0·0 (NS)	0·1 (NS)	−0·3 (NS)	−0·0 (NS)
TZEEI 88	TZEF-YSR BC1 X 9450 STR S6inb 42-2-2	159 (NS)	1·9 (*P*< 0·01)	1·7 (*P*< 0·01)	−0·2 (NS)	1 (NS)	7 (*P*< 0·05)	−0·3 (NS)	−0·0 (NS)	0·1 (NS)	0·0 (NS)
TZEEI 89	TZEF-YSR BC1 X 9450 STR S6inb 13A	−168 (NS)	0·0 (NS)	−0·4 (NS)	−0·3 (NS)	−9 (*P*< 0·01)	−4 (NS)	0·1 (NS)	0·1 (NS)	−0·1 (NS)	−0·0 (NS)
TZEEI 94	TZEE-Y Pop Co S6 Inbred 47-2-4B	−525 (NS)	0·1 (NS)	−0·1 (NS)	−0·2 (NS)	−2 (NS)	2 (NS)	−0·1 (NS)	0·1 (NS)	0·5 (NS)	−0·0 (NS)
TZEEI 95	TZEE-Y Pop Co S6 Inbred 47-3-4	−55 (NS)	−0·4 (*P*< 0·01)	−0·6 (*P*< 0·01)	−0·2 (NS)	−7 (*P*< 0·01)	−2 (NS)	0·2 (NS)	0·1 (*P*< 0·05)	−0·1 (NS)	0·0 (*P*< 0·05)
TZEEI 96	TZEE-Y Pop Co S6 Inbred 78	−413 (NS)	1·2 (*P*< 0·01)	1·4 (*P*< 0·01)	0·1 (NS)	2 (NS)	2 (NS)	−0·0 (NS)	0·1 (NS)	0·3 (NS)	−0·0 (NS)
TZEEI 97	TZEE-Y Pop Co S6 Inbred 101-1-2	338 (NS)	−0·7 (*P*< 0·05)	−0·9 (*P*< 0·05)	−0·2 (NS)	56 (NS)	3 (NS)	0·0 (NS)	0·0 (NS)	0·5 (NS)	0·0 (NS)
TZEEI 98	TZEE-Y Pop Co S6 Inbred 101-2-4	3649 (*P*< 0·01)	−0·4 (NS)	−0·5 (NS)	−0·1 (NS)	7 (*P*< 0·05)	5 (NS)	−0·2 (NS)	−0·1 (NS)	0·3 (NS)	0·0 (NS)
TZEEI 99	TZEE-Y Pop Co S6 Inbred 101-1-4	−15 (NS)	−0·2 (NS)	−0·3 (NS)	−0·1 (NS)	11 (*P*< 0·01)	9 (*P*< 0·01)	−0·3 (NS)	−0·0 (NS)	0·1 (NS)	0·0 (*P*< 0·05)
TZEEI 100	TZEF-Y POP STR COS6 inb 47-3-4	385 (NS)	0·8 (*P*< 0·05)	0·8 (NS)	−0·0 (NS)	2 (NS)	6 (*P*< 0·05)	−0·2 (NS)	0·2 (NS)	0·7 (NS)	0·0 (NS)
TZEEI 101	TZEF-Y POP STR COS6 inb 101-1-4	−70 (NS)	−0·2 (NS)	0·1 (NS)	0·3 (NS)	−2 (NS)	−1 (NS)	1·8 (*P* < 0·01)	0·2 (NS)	−0·3 (NS)	0·0 (NS)
TZEEI 102	TZEF-Y SR BC1 X 9450 STRS6 inb 7A	−73 (NS)	0·1 (NS)	0·3 (NS)	0·2 (NS)	−7 (*P* < 0·05)	−11 (*P* < 0·01)	0·2 (NS)	0·0 (NS)	−0·2 (NS)	−0·1 (*P* < 0·01)
TZEEI 108	TZEF-Y SR BC1 X 9450 STR inb 7-1-2A	308 (NS)	0·4 (NS)	1·1 (*P* < 0·01)	0·7 (*P* < 0·01)	6 (NS)	4 (NS)	−0·2 (NS)	−0·1 (NS)	0·2 (NS)	−0·0 (NS)
TZEEI 115	TZEF-Y SR BC1 X 9450 STR inb 5	−59 (NS)	0·2 (NS)	0·4 (NS)	0·2 (NS)	−3 (NS)	−5 (NS)	0·0 (NS)	−0·2 (*P* < 0·05)	0·2 (NS)	−0·0 (NS)
S.E.		595·4	0·36	0·43	0·19	3·2	3·0	0·31	0·10	0·50	0·020
Proportion of GCA over SCA		0·48	0·70	0·74	0·69	0·81	0·84	0·56	0·71	0·45	0·60

DYSK, days to silk; ASI, anthesis-silking interval; PLHT, plant height; EHT, ear height; PASP, plant aspect; EASP, ear aspect; EPP, ears per plant; HC, husk cover; GCA, general combining ability; SCA, specific combining ability; NS, not significant; S.E., standard error between two GCA effects.

Under drought, significant (*P* < 0·05) and positive GCA effects for grain yield, ear height and ears per plant were observed for inbred TZEEI 97, while TZEEI 63 had significant (*P*< 0·01) and negative GCA effects for grain yield, plant height, ear height and ears per plant (Table 5). However, significant (P < 0·05) and positive GCA effects for DA, days to silking, anthesis-silking interval, plant aspect and ear aspect were observed for inbreds TZEEI 62 and TZEEI 63 under drought environments. In contrast, TZEEI 98 had significant (*P*< 0·01) and positive GCA effects for grain yield under rain-fed conditions (Table 6).

Heterotic groupings of the extra-early yellow inbred lines under drought

The SCA effects and mean grain yields were used as the basis for classifying the extra-early maize inbred lines into heterotic groups. To belong to a heterotic group, a line must have significant (*P*< 0·05) positive SCA effects with one of the testers and significant (*P* < 0·05) negative SCA effects with the other, as well as a mean yield ⩾1 standard error (S.E.) above the grand mean of all test-crosses involving one of the positive SCA testers (Agbaje *et al*. 2008). Lines that had zero SCA effects were not classified into any heterotic group. Among the 39 inbred lines with positive SCA effects for grain yield, only 16 had significant effects (Table 4). Two inbreds were classified into TZEEI 63 heterotic group, one into TZEEI 79 and three into TZEEI 95 heterotic groups (Table 7). The two inbreds (TZEEI 71 and TZEEI 102) classified into the TZEEI 63 heterotic group had significant negative SCA effects with this tester but positive SCA effects with TZEEI 79 and TZEEI 95 and grain yield of at least 1981 and 2707 kg/ha; which is >1 S.E. and higher than 1776 and 1756 kg/ha, respectively. In contrast, the three inbreds classified into the TZEEI 95 heterotic group had a significant (*P* < 0·05) and negative SCA effects with this tester; two of the three lines (TZEEI 99 and TZEEI 101) had significant (*P* < 0·01) and positive effects with TZEEI 63 and the remaining one had significant (*P* < 0·05) and positive effects with TZEEI 79 with grain yield of at least 1713 kg/ha which is >1 S.E. and higher than 1199 kg/ha (Table 7).

**Table 7:** Grain yield and specific combining ability (SCA) effects of 18 out of 39 extra-early inbreds evaluated in testcrosses with three testers under induced drought conditions at Ikenne in 2010 and 2011, terminal drought at Bagauda in 2010

LINE	Mean grain yield (kg/ha)		SCA effects with		
	TZEEI 63	TZEEI 79	TZEEI 95	TZEEI 63	TZEEI 79	TZEEI 95
TZEEI 60	1066	1373	3987	−48	−319	2316 (*P*< 0·01)
TZEEI 61	1111	1686	2434	−168	−170	598 (*P*< 0·01)
TZEEI 62	503	1486	1993	−446	−40	488 (*P*< 0·01)
TZEEI 69	1616	1729	1636	335 (*P*< 0·05)	−130	−203
TZEEI 71	922	2253	1981	−4188 (*P*< 0·01)	336 (*P*< 0·05)	84
TZEEI 72	935	1948	1338	−93	342 (*P*< 0·05)	−247
TZEEI 74	728	2055	1438	−300	449 (*P*< 0·01)	−148
TZEEI 76	761	1960	1853	−385 (*P*< 0·05)	236	151
TZEEI 77	1337	1311	1245	418 (*P*< 0·05)	−185	−231
TZEEI 78	1446	1501	1457	357 (*P*< 0·05)	−166	−189
TZEEI 87	1652	1419	1503	506 (*P*< 0·01)	−304	−200
TZEEI 96	1664	1037	2009	473 (*P*< 0·01)	−732 (*P*< 0·01)	260
TZEEI 97	1551	2471	1637	44	386 (*P*< 0·05)	−428 (*P*< 0·01)
TZEEI 98	1982	1756	1665	561 (*P*< 0·01)	−244	−315
TZEEI 99	1826	1512	1382	632 (*P*< 0·01)	−260	−370 (*P*< 0·05)
TZEEI 101	1743	1908	1487	409 (*P*< 0·01)	−3	−404 (*P*< 0·05)
TZEEI 102	462	1516	2707	−578 (*P*< 0·01)	−101	1110 (*P*< 0·01)
TZEEI 115	728	1833	1885	−375 (*P*< 0·05)	152	225
Mean*	1199	1776	1756	0	0	0
Standard error	101·0	103·0	104·0	174·5	174·5	174·5

* Mean grain yield of 39 inbred lines.

### Biplot analysis

**Fig. 1:**
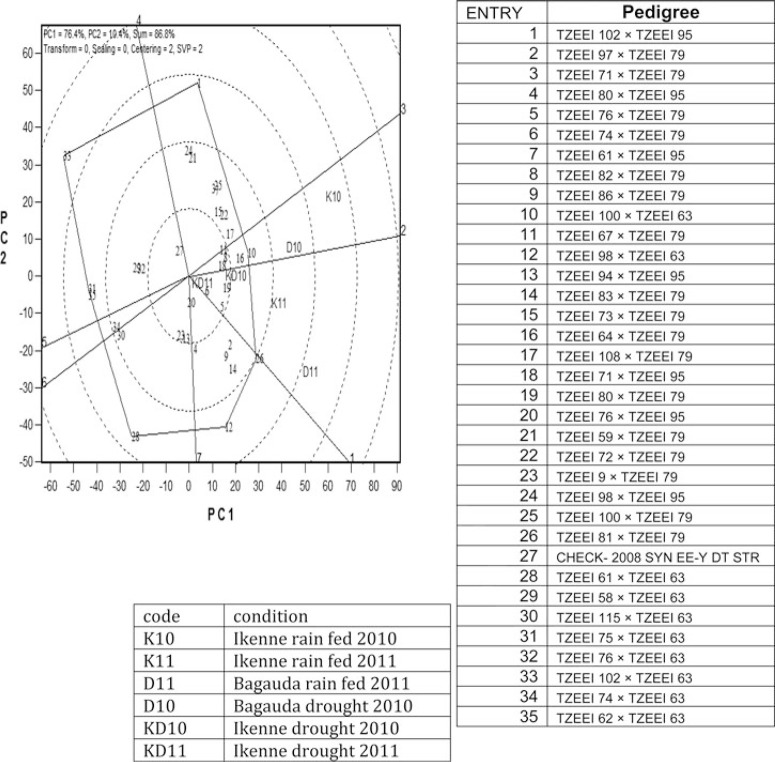
A ‘which won where’ genotype plus genotype × environment interaction biplot of grain yield of 35 extra-early maturing maize hybrids evaluated at six locations; three under drought (Ikenne and Bagauda) and three under rain-fed conditions (Ikenne and Bagauda) in 2010 and 2011. The biplot was based on genotype-focused singular value partitioning (‘SVP = 2’) and is therefore appropriate for visualizing the relationship among test sites. Principal component (PC) 1 and PC2 for model 3 explained 86% of the yield variation.

**Fig. 2:**
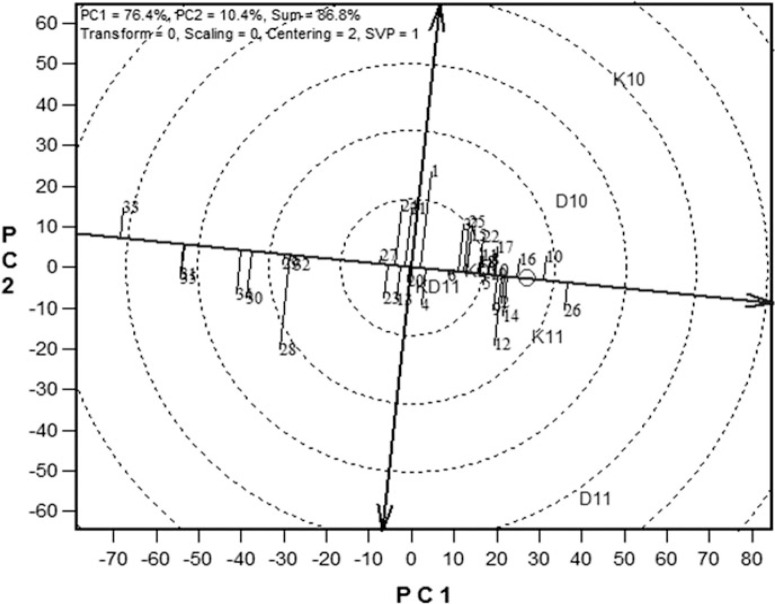
The entry/tester genotype plus genotype × environment biplot based on grain yield of 35 extra-early maturing maize hybrids evaluated at six locations; three under drought (Ikenne and Bagauda) and three under rain-fed conditions (Ikenne and Bagauda) in 2010 and 2011. The biplot was based on environment-focused singular value partitioning (‘SVP = 1’) and is therefore appropriate for visualizing the relationship among genotypes. Principal component (PC) 1 and PC2 for model 3 explained 86% of the yield variation. See Fig. 1 for hybrid and site legends.

The highly significant (*P*< 0·01) GEI for grain yield and most measured traits under drought and rain-fed conditions justified the use of the GGE biplot to decompose the GEI and to examine the yield performance and stability of the extra-early hybrids across research environments. The GGE biplot for grain yield of the 35 (26 best and nine worst) extra-early maturing maize hybrids evaluated at six locations across two research conditions is shown in Figs 1 and 2. In the polygon view (Fig. 1), the vertex hybrid represents the highest yielding hybrid in the location that falls within the sector. TZEEI 100 × TZEEI 63 (entry 10) and TZEEI 64 × TZEEI 79 (entry 16) were the highest-yielding hybrids at Ikenne rain-fed and Bagauda drought in 2010 while the hybrid TZEEI 81 × TZEEI 79 (entry 26) was highest-yielding at Ikenne under drought conditions in 2010 and 2011, and at Bagauda and Ikenne under rain-fed conditions in 2011 (Fig. 1). The vertex hybrids TZEEI 102 × TZEEI 95 (entry 1), TZEEI 98 × TZEEI 63 (entry 12), TZEEI 75 × TZEEI 63 (entry 31), TZEEI 102 × TZEEI 63 (entry 33) and TZEEI 62 × TZEEI 63 (entry 35) were the highest-yielding in their sectors. Furthermore, some sectors contained no locations, indicating that these hybrids were not the best in any of the test environments under drought or rain-fed conditions. Hybrids within the polygon, particularly those located close to the biplot origin, were less responsive than the vertex hybrids.

In the GGE biplot presented in Fig. 2, the single-arrow line that passes through the biplot origin and the average environment is referred to as the average-tester axis; the black arrow points to the average environment from the biplot origin. The hybrids were ranked along the average-tester axis, with the arrow pointing to a greater value based on their mean performance across testing sites; the double arrow line average tester coordinate (ATC) separates the entries with below-average means from those with above-average means. The average yield of the hybrids is estimated by the projections of their markers on the average-tester axis. The stability of the hybrids is measured by their projection onto the average-tester coordinate y-axis single-arrow line (ATC abscissa). The greater the absolute length of the projection of a hybrid, the less stable it is. Thus, TZEEI 81 × TZEEI 79 (entry 26), TZEEI 100 × TZEEI 63 (entry 10) and TZEEI 64 × TZEEI 79 (entry 16) were the most stable hybrids, followed by Entries 2 (TZEEI 97 × TZEEI 79), 9 (TZEEI 86 × TZEEI 79), 14 (TZEEI 83 × TZEEI 79) and 17 (TZEEI 108 × TZEEI 79), while entry 1 (TZEEI 102 × TZEEI 95) was the least stable. Entry 26 was the highest-yielding hybrid, while TZEEI 102 × TZEEI 63 (entry 33) was the lowest.

## DISCUSSION

The significant mean squares observed for the hybrids for all traits under drought and rain-fed conditions indicated that there was adequate genetic variation among the hybrids for the measured traits to allow good progress from selection for tolerance to drought at flowering and grain filling. The significant genotype × environment interaction obtained in the present study provided a justification for the strategy of evaluating entries across two distinct test environments in an effort to identify hybrids with consistent performance across the environments. These results are consistent with the findings of Badu-Apraku *et al*. (2011) and Badu-Apraku & Oyekunle (2012). The highly significant mean squares for E, E × GCA of lines and E × GCA of testers for most traits measured under drought indicated that the combining ability of most lines and testers varied under the test environments. According to Scott (1967), testing lines under different environments should facilitate the selection of stable testers and hybrids. The significant SCA × E interactions observed for grain yield and most measured traits under drought and rain-fed conditions implied that the yield performance of the hybrids was not consistent in the varying environments. The implication is that GEI effects might create difficulties in selection for the required traits in different environments. This result provided a justification for the strategy of evaluating hybrids across two test environments in an effort to identify those with a consistent performance across the contrasting environments. Furthermore, this finding suggested the need for evaluating the hybrids in contrasting environments under managed drought and terminal drought conditions to identify those with consistent favourable responses, for the development of drought-tolerant maize hybrids. This result is also consistent with the findings of several authors (Badu-Apraku *et al*. 2011; Badu-Apraku & Oyekunle 2012). The lack of significant E × SCA interactions means squares for days to silking, anthesis-silking interval, ear height, ear aspect and ears per plant under drought and E × GCA for anthesis-silking interval and plant aspect and E × SCA for plant aspect under rain-fed conditions indicated that the hybrids expressed these traits consistently in different environments. The highly significant GCA and SCA mean squares for grain yield and most other traits under both research conditions indicated that both additive and non-additive gene actions were important in the inheritance of most measured traits. Under drought, GCA accounted for 72·7% of the total variance for yield indicating that additive gene action largely controlled the inheritance of yield of the hybrids. In contrast, SCA accounted for 51·6% of the total variance for grain yield under rainfed conditions, indicating that non-additive gene action largely controlled the inheritance of grain yield of the hybrids under rain-fed conditions. This implies that appreciable breeding progress could be made using breeding methods which capitalize on additive gene action such as the S1 family recurrent selection, backcrossing and hybridization for the development of drought-tolerant cultivars, and synthetics as well as for population improvement. This result is in agreement with the findings of Vasal *et al*. (1992) and Zambezi *et al*. (1994).

In the present study, the yield reduction of 52% under drought indicated that the levels of droughtimposed during flowering and grain-filling were severe enough to allow selection for drought tolerance among the hybrids. The yield reduction observed under drought was within the range reported by other researchers (NeSmith & Ritchie 1992; Badu-Apraku *et al*. 2011). Akaogu *et al*. (2012) evaluated the same set of hybrids under *Striga*-infested and *Striga*-free environments and reported mean grain yield of 1·69 t/ha and 2·78 t/ha, respectively, with a yield reduction of 37% under *Striga*-infested conditions. The implication is that drought stress caused greater yield loss than *Striga*-infestation in this set of hybrids.

The significant positive GCA effects for grain yield observed for the extra-early yellow inbreds TZEEI 97 and TZEEI 98 under drought and rain-fed conditions indicated that these inbreds could be invaluable sources of favourable alleles for breeding for improved grain yield under these conditions. The lines with favourable GCA effects for grain yield and other traits are expected to transmit their characteristics to the offspring hence they could be used as parental lines to develop synthetic populations or outstanding hybrids for drought-prone areas in the tropics.

A good tester is expected to give the most precise classification among genotypes. Hallauer (1975) further showed that a tester with low gene frequency for the trait being classified would give greater variances in the partial-to-complete dominance range. The results of the present study indicated that the above characteristics of good testers are confusing and contradictory and that a line with above average GCA is not necessarily a good tester (I.C. Akaogu, personal communication, 2011). The testers used in the present study did not meet all the criteria required for a good tester and, therefore, could not classify several of the extra-early inbred lines into well-defined heterotic groups. Nevertheless, the inbreds classified into the three heterotic groups may be inter-crossed separately to develop three complementary populations for improvement using recurrent selection. In the present study, some inbreds derived from the same source population were classified into different heterotic groups, confirming the broad genetic diversity of the source populations from which the lines used in the study were extracted. The inbred lines could, therefore, be regarded as having been derived from diverse genetic backgrounds. This was expected since the source populations were developed from mixtures of different genetic composition (Badu-Apraku & Oyekunle 2012).

Under drought stress, grain yield was positively correlated with ears per plant, plant and ear height but negatively correlated with the flowering traits (DA and silking), plant aspect, ear aspect, husk cover and the stay green characteristic. These findings are consistent with the results of Bolanos & Edmeades (1996). The decrease in grain yield under drought stress was associated with a reduction in the anthesis-silking interval, barrenness, stay green character, poor plant and ear aspects. Bolanos *et al*. (1993) and Edmeades *et al*. (1995) reported that ears per plant and anthesis-silking interval are important secondary traits when selecting for drought tolerance and yield potential in tropical maize. The means of the flowering traits were larger under drought stress than under optimal growing conditions. In contrast, the means for vegetative traits (plant height and ear height) were higher under rain-fed conditions than under drought stress. Thus, production traits were more susceptible to drought stress than the vegetative traits. Furthermore, the coefficients of variation (CVs) for grain yield and most measured traits associated with drought stress were consistently larger than those under rain-fed conditions. In the present study, the drought stress induced from three WAP till physiological maturity was adequate to elicit the genetic variation among the hybrids.

In WCA, the naturally available mechanisms for drought escape and drought tolerance in the maize germplasm and the prevailing production environments have been exploited to develop cultivars with enhanced adaptation to stressful environments. Drought escape occurs when the plant completes critical physiological processes before drought sets in. This trait is desirable in cultivars intended for release to farmers in areas where terminal drought is most prevalent. In contrast, adaptation to drought-prone environments is under genetic control and indicates the presence of physiological mechanisms that minimize or withstand the adverse effects of drought if and when it occurs. Cultivars with enhanced adaptation to drought-prone environments are useful where drought occurs randomly and at any growth stage of the maize crop. This is relevant in WCA where drought occurrence is erratic, with varying timing and levels of intensity. Using the two strategies, during the past two decades IITA has developed a wide range of high-yielding drought-tolerant or drought-escaping extra-early cultivars to combat the threat posed by recurrent drought in the savannas of WCA. The outstanding performance of many extra-early hybrids compared to the open-pollinated control variety in the present study is a clear indication of the considerable progress that has been made in breeding for high-yielding, drought-tolerant extra-early maize hybrids for the savannas of SSA. The high repeatability estimates (>0·78) observed for all measured traits under each and across research conditions suggested that the performance of the hybrids would be readily reproducible.

Although it would have been more appropriate to use a drought-tolerant extra-early yellow hybrid as control variety for comparison with the extra-early single-cross hybrids evaluated in the current study, no extra-early hybrid with drought tolerance at the flowering and grain-filling periods was available when the study began. Consequently, the best drought-tolerant and *Striga*-resistant extra-early maturing open-pollinated variety available in the IITA Maize Program, 2008 Syn EE-Y DT STR was used as the control.

Using the base index, TZEEI 83 × TZEEI 79, TZEEI 80 × TZEEI 79, TZEEI 100 × TZEEI 63, TZEEI 108 × TZEEI 79 and TZEEI 100 × TZEEI 79 were identified as *Striga*-resistant hybrids (Akaogu *et al*. 2012).These hybrids were also identified to be drought-tolerant in the present study using the base index. The identification of these five extra-early hybrids as resistant to *Striga* and also tolerant to drought is expected to contribute to increased maize production and productivity in SSA, since the two stresses occur simultaneously under field conditions and the combined effect can be devastating (Cechin & Press 1994; Kim & Adetimirin 1997). Therefore, the outstanding hybrids should be further tested in on-farm trials under drought, *Striga* and rain-fed environments to confirm the consistency of performance for commercialization in SSA.

The GGE biplot identified TZEEI 81 × TZEEI 79 (entry 26), TZEEI 100 × TZEEI 63 (entry 10) and TZEEI 64 × TZEEI 79 (entry 16) as the highest-yielding and most stable hybrids across research conditions. These hybrids are recommended for on-farm testing, especially in the environments in which they showed superiority, a requirement for varietal release and commercialization in WCA, and these should be tested on-farm for commercialization in the sub-region. Furthermore, the hybrid TZEEI 64 × TZEEI 79 had high grain yield and was the closest to the ideal genotype and may be considered as the ideal hybrid in the present study. On the other hand, the vertex hybrids TZEEI 100 × TZEEI 63 (entry 10) and TZEEI 64 × TZEEI 79 (entry 16) were the highest-yielding hybrids at Ikenne under rain-fed conditions and Bagauda under drought in 2010, while TZEEI 81 × TZEEI 79 (entry 26) was the highest-yielding at Ikenne under drought conditions in 2010 and 2011, and at Bagauda and Ikenne under rain-fed conditions in 2011. These hybrids should be tested extensively in these environments to confirm the consistency of performance prior to release in those areas.

In conclusion, the extra-early drought-tolerant hybrids TZEEI 81 × TZEEI 79, TZEEI 64 × TZEEI 79 and TZEEI 100 × TZEEI 63 were identified in the present study as possessing drought-tolerance genes and would tolerate drought that occurs at the flowering and grain-filling periods, in addition to their drought-escaping ability (a characteristic of extraearliness). Lastly, the results of the present study have confirmed that drought stress induced from 3 WAP till physiological maturity was adequate to elicit the genetic variation among the hybrids.

The authors are grateful for financial support from Alliance for a Green Revolution in Africa (AGRA), Drought Tolerant Maize for Africa (DTMA) and the International Institute of Tropical Agriculture (IITA), Ibadan. Also, the technical assistance from the staff of Maize Improvement Unit of IITA, Ibadan is highly appreciated.
